# The relationship between oxidised LDL, endothelial progenitor cells and coronary endothelial function in patients with CHD

**DOI:** 10.1136/openhrt-2015-000342

**Published:** 2016-01-28

**Authors:** Jonathan Watt, Simon Kennedy, Nadeem Ahmed, James Hayhurst, John D McClure, Colin Berry, Roger M Wadsworth, Keith G Oldroyd

**Affiliations:** 1 West of Scotland Regional Heart & Lung Centre, Golden Jubilee National Hospital, Glasgow, UK; 2 Institute of Cardiovascular and Medical Sciences, University of Glasgow, Glasgow, UK; 3 Strathclyde Institute of Pharmacy and Biomedical Sciences, University of Strathclyde, Glasgow, UK

**Keywords:** CORONARY PHYSIOLOGY

## Abstract

**Objective:**

The balance between coronary endothelial dysfunction and repair is influenced by many protective and deleterious factors circulating in the blood. We studied the relationship between oxidised low-density lipoprotein (oxLDL), circulating endothelial progenitor cells (EPCs) and coronary endothelial function in patients with stable coronary heart disease (CHD).

**Methods:**

33 patients with stable CHD were studied. Plasma oxLDL was measured using ELISA, coronary endothelial function was assessed using intracoronary acetylcholine infusion and EPCs were quantified using flow cytometry for CD34^+^/KDR^+^ cells.

**Results:**

Plasma oxLDL correlated positively with the number of EPCs in the blood (r=0.46, p=0.02). There was a positive correlation between the number of circulating EPCs and coronary endothelial function (r=0.42, p=0.04). There was no significant correlation between oxLDL and coronary endothelial function.

**Conclusions:**

Plasma levels of oxLDL are associated with increased circulating EPCs in the blood of patients with CHD, which may reflect a host-repair response to endothelial injury. Patients with stable CHD had a high prevalence of coronary endothelial dysfunction, which was associated with lower numbers of circulating EPCs, suggesting a mechanistic link between endothelial dysfunction and the pathogenesis of atherosclerosis.

**Key questions**

**What is already known about this subject?**

- Endothelial progenitor cells (EPCs) are crucial mediators of vascular repair following endothelial injury and the number of circulating EPCs is influenced by many factors, including myocardial ischaemia and levels of proinflammatory cytokines.

**What does this study add?**

- We found the number of circulating EPCs was correlated with plasma oxidised low-density lipoprotein (oxLDL) in patients with stable coronary heart disease, most of whom were taking statins and other cardioprotective medications. The number of circulating EPCs also correlated with a measure of coronary endothelial function.

**How might this impact on clinical practice?**

- Our study suggests a host-repair response to oxLDL, demonstrated by an increase in circulating EPCs. Elevated levels of EPCs have the potential to improve endothelial function. Our data highlight the potential role of targeted measures to promote endothelial function which, in turn, may improve cardiovascular health.

## Introduction

Oxidised low-density lipoprotein (oxLDL) injures the vascular endothelium, a key step in the pathogenesis of atherosclerosis.^1^ An important mechanism of endothelial repair involves the mobilisation and homing of bone marrow-derived endothelial progenitor cells (EPCs) to sites of injury, repopulating the artery with functional endothelial cells (ECs). Circulating levels of EPCs in blood are increased following many forms of endothelial injury including ischaemia in experimental animals^2^ and myocardial infarction in humans,^3^ ^4^ but there are conflicting data on whether circulating EPC levels correspond to the severity of coronary heart disease (CHD).^5^ ^6^ EPCs are more resistant than mature ECs to the toxic effects of oxidative stress due to greater expression of potent antioxidant enzymes^7^ that allow EPCs to proliferate and differentiate in areas of increased oxidative stress, such as ischaemic tissues. EPC mobilisation is known to be triggered by several proinflammatory cytokines and growth factors including granulocyte macrophage-colony stimulating factor, stromal cell-derived factor-1, matrix metalloproteinases, vascular endothelial growth factor (VEGF) and erythropoetin.^8^ In laboratory studies, oxLDL is toxic to cultured EPCs,^9–12^ however, the in vivo relationship between plasma oxLDL and the number of circulating EPCs in the blood is not known. Lowering oxLDL in humans pharmacologically has a heterogeneous effect on endothelial dysfunction, with most coronary segments showing enhancement of dilation to acetylcholine (ACh), but other segments showing a reduction in dilation.^13^ We hypothesised that elevated plasma oxLDL in patients with stable CHD would result in an increase in circulating EPCs and that this may have a positive effect on endothelial function. The aim of this study was therefore to investigate the relationship between plasma oxLDL and EPCs in patients with stable CHD and whether circulating EPCs influence coronary endothelial function. To our knowledge, this is the first study to investigate this relationship and our results provide evidence that elevated oxidative stress may stimulate mechanisms involved in endothelial repair, thereby exerting an effect on endothelial function.

## Methods

### Study population

This cross-sectional observational study was approved by the West Glasgow ethics committee (05/S0709/138-Coronary stent deployment, oxidative stress, endothelial regeneration and risk of thrombosis) and the investigation conformed with the principles outlined in the Declaration of Helsinki. All individual data were collected in a blinded fashion. All participants were provided with a Patient Information Sheet and gave informed written consent. To be included in the study, patients had to be over age 18 and scheduled to undergo elective percutaneous coronary intervention (PCI) to treat stable CHD. All patients had at least one major epicardial coronary artery suitable for endothelial function testing (<30% stenosis and at least 2.5 mm in diameter). Patients were excluded if there was a history of myocardial infarction (MI) within 3 months or if they were unable to give informed consent. One hour before PCI, venous blood was removed for immediate assay of circulating EPCs and plasma was stored at −70°C for subsequent oxLDL assay. Patients then underwent routine cardiac catheterisation as planned with the additional component of invasive coronary endothelial function testing prior to PCI.

### OxLDL assay

OxLDL was detected by the commercial Mercodia solid two-site ELISA (Diagenics, Bletchley, UK). In this assay, two monoclonal antibodies were directed against separate antigenic determinants on the oxidised apolipoprotein B molecule. Samples were snap-thawed at 37°C for 3 min. All samples were run in duplicate. The average coefficient of variation of the duplicates was 0.6%. The lowest sample value was 36 IU, which was above the lowest standard (10 IU). All samples were therefore within the sensitivity of the assay.

### EPC assay

EPC sample preparation involved 1 mL of EDTA anticoagulated blood added to 100 µL of Fc receptor (FcR) blocking reagent (Miltenyi Biotec) and incubated for 20 min at room temperature. FcR blocking reagent stopped the non-specific binding of IgG, reducing the background noise in the sample. Five tubes were then prepared, numbered 1–5, with 20 µL of anti-VEGF receptor-2 APC added to tubes 1,3,4,5 and 20 µL IgG1-APC was added to tube 2. One hundred microlitres of the blocked blood, containing the FcR blocking reagent, was then added to each of the tubes (1–5). All tubes were incubated for a further 30 min on ice and protected from light. Twenty microlitres of 7-amino-actinomycin D visibility dye was added to each tube for the exclusion of non-viable cells. Twenty microlitres of CD45-FITC/CD34-PE antibody combination was then added to tubes 2, 3, 4, 5, with 20 µL of CD45-FITC/CTL-PE added to tube 1. These tubes were incubated at room temperature for 20 min and protected from light. Essentially, tube 1 acted as a control for CD34 using CD45-FITC/CTL-PE and tube 2 as a control for KDR, by adding CD45-FITC/CD34-PE. Diluted lysis buffer was made up by adding 400 µL 10× buffer to 3.6 mL of water. After 20 min incubation, 500 µL of lysis buffer was added to each tube before a further 10 min of incubation in the dark. Finally, 100 µL of a fluorosphere stem count reagent was added to each tube to prepare the sample for fluorescence-activated cell sorting (FACS) acquisition.

EPCs were quantified by flow cytometry using FACS analysis to look for markers CD34 and KDR, thought to define EPCs.^14^ Data were analysed using Cellquest Pro Software (BD) which involved the use of analytical gates to count the total number and subsets of circulating cells. Samples were run until 250 000 events were counted or 20 min had elapsed. Data were then stored for future analysis where EPCs were quantified as ((the number of CD34^+^KDR^+^) events)×(concentration of fluorospheres))/(total number of fluorospheres) per μL of whole blood.

### Coronary endothelial function testing

All vasodilator medications were withheld for 24 h prior to testing. Coronary endothelial function was measured by the standard method of assessing the change in luminal diameter, using quantitative coronary angiography (QCA), in response to an intracoronary infusion of the endothelium-dependent vasodilator, ACh. In healthy arteries, ACh causes release of nitric oxide (NO) from ECs, leading to vasodilation. In endothelial dysfunction, the NO response is blunted and the direct muscarinic smooth muscle response to ACh predominates, causing paradoxical vasoconstriction. Following baseline coronary angiography, the optimal angiographic projection for the study artery was selected, avoiding overlapping side-branches. A 3 French infusion catheter (Cook Medical, Limerick, Ireland) was placed into the proximal portion of the artery to be studied via a standard 6 French guiding catheter. After intracoronary infusion of 0.9% saline as a control, endothelium-dependent vasomotion was assessed by serial infusions of ACh (Miochol-E, Novartis) in the following order: 10^−6^, 10^−5^ and 10^−4^ M at a flow rate of 2 mL/min for 2 min. Assuming a mean coronary artery blood flow of 50 mL/min, the final blood concentration for each ACh infusion was 4×10^−8^, 4×10^−7^ and 4×10^−6^ M, which was appropriate for the assessment of vasomotor responses (recommended range 10^−8^ to 10^−5^ M).^15^ At the end of each infusion, coronary angiography was performed with contrast medium (Omnipaque, GE Healthcare) using identical projections, table height and magnification. The ACh infusion was terminated if significant ischaemia or bradycardia were observed. Endothelium-independent vasomotion was finally assessed using an intracoronary bolus injection of 400 µg isosorbide dinitrate. If required, a further dose of isosorbide dinitrate was administered to fully reverse any latent effects of ACh, ensuring maximal coronary artery vasodilation before a final angiogram was recorded.

### Quantitative coronary angiography

Coronary angiograms were stored digitally at the Golden Jubilee National Hospital, Glasgow for subsequent analysis. An automated edge-detection software system (Centricity CA1000, GE Healthcare) was used to measure the luminal diameter of the coronary artery distal to the infusion catheter, at end diastole. The mean percentage change in luminal diameter compared to control in two distinct 5 mm segments was calculated for each patient following each infusion and after final nitrate injection (each analysis segment consisted of five separate measurements 1 mm apart). Each segment was easily identifiable by anatomical landmarks (such as side branches). Endothelial function was defined by the vasomotor response (% change in luminal diameter) to the highest tolerated ACh infusion, compared to control. Negative changes in vessel diameter represent vasoconstriction. One patient did not receive 10^−4^ M ACh due to recurrent atrioventricular block. All other patients received all three concentrations of ACh. All QCA analysis was performed by a single observer, blinded to all other study results.

### Statistical analysis

A sample size of 25 patients was estimated to provide 80% power to detect a relationship between the independent and the dependent variables at a two-sided 0.05 significance level, if the true change in the dependent variable is 0.3 units per unit change in the independent variable. This was based on the assumption that the SD of the independent variable (oxLDL) is 0.3 and the SD of the dependent variable (endothelial function) is 0.15.^16^ All data are expressed as mean±SEM unless otherwise stated. Correlations were performed using the Pearson correlation coefficient. To check whether other patient characteristics influenced the relationship between EPCs and oxLDL, gender, hypertensive status and hypercholesterolaemic status were each individually added to the model. Coronary responses for each infusion were compared using analysis of variance and post hoc Dunnett's test. Statistical analysis was performed using the SPSS statistical software package V.14.0 for Windows (SPSS Inc, Chicago, Illinois, USA).

## Results

### Study participants

In total, 33 patients consented to the study. The endothelial function study was not performed in one patient due to time constraints in the catheterisation laboratory and in another patient due to myocardial ischaemia related to deep engagement of the infusion catheter. Results for oxLDL (2 patients) and EPCs (8 patients) were not available for analysis due to machine breakdown or late sample arrival. The patient baseline characteristics are shown in table 1. Individual patient data of plasma oxLDL concentration, EPC count and endothelial function are shown in table 2. All patients were receiving oral aspirin and clopidogrel therapy along with standard pharmacotherapy for CHD, including a high prevalence of statins.

**Table 1:** Baseline characteristics of patients

Baseline characteristics of patients	Prevalence (n=33)
Age, mean±SD (years)	62.4±8.36
Clinical characteristics, n (%)	
Male	25 (75.8)
Female	8 (24.2)
Hypertension	17 (51.5)
Hypercholesterolaemia	23 (69.7)
Diabetes mellitus	3 (9.1)
Current smoker	4 (12.1)
Positive family history for CHD	16 (48.5)
Single vessel disease	26 (78.8)
Previous MI	9 (27.3)
Previous stroke	1 (3.03)
Preserved LV function	32 (97.0)
Heart failure	0 (0.0)
Previous PCI or CABG	3 (9.1)
Drug treatment, n (%)	
Aspirin	33 (100.0)
Clopidogrel	33 (100.0)
ACE inhibitor	17 (51.5)
Angiotensin receptor blocker	4 (12.1)
β-blocker	29 (87.9)
Calcium channel blocker	13 (39.4)
Diuretic	16 (48.5)
Nitrate	16 (48.5)
Nicorandil	6 (18.2)
Statin	30 (90.9)

CHD, coronary heart disease; PCI, percutaneous coronary intervention; MI, myocardial infarction; LV, left ventricular; CABG, coronary artery bypass graft.

**Table 2:** Individual patient results for oxLDL, EPCs and coronary response to ACh

Patient No	oxLDL (IU)	EPCs (µL blood)	% Change in diameter (10^−4^ M ACh)
1	36.34	0.2672	−1.6140
2	70.33	0.0142	−18.2796
3	49.39	0	−34.1583
4	100.89	0.3351	14.1741
5	82.65	0.3233	−0.2766
6	72.66	0.4110	−14.7591
7	65.92	1.1996	−2.1605
8	72.08	0.5567	3.3432
9	56.38	0.2376	−8.6903
10	56.06	ND	−6.1734
11	44.22	0.4284	−1.6225
12	42.87	0.4909	−2.3068
13	38.77	0	−37.7922
14	53.55	0.2537	−47.2260
15	23.36	0.0480	−37.0728
16	42.52	ND	−8.5830
17	20.01	ND	−17.5202
18	36.22	0	−14.9939
19	41.86	ND	−12.1033
20	34.64	0.0976	−19.6740
21	NS	0.0505	−26.7600
22	48.35	0.5088	3.7042
23	42.25	ND	−3.9413
24	36.81	0	16.5367
25	29.26	0	−19.0864
26	29.33	0	ND
27	41.92	0.0788	−11.3369
28	41.01	0	−3.9623
29	41.73	0.0266	−14.1770
30	26.31	ND	ND
31	33.99	ND	−1.5818
32	NS	ND	−9.2091
33	50.65	0	−39.1786

ACh, acetylcholine; EPCs, endothelial progenitor cells; ND, not determined; NS, no sample; oxLDL, oxidised low-density lipoprotein.

### Coronary endothelial function

**Figure 1:**
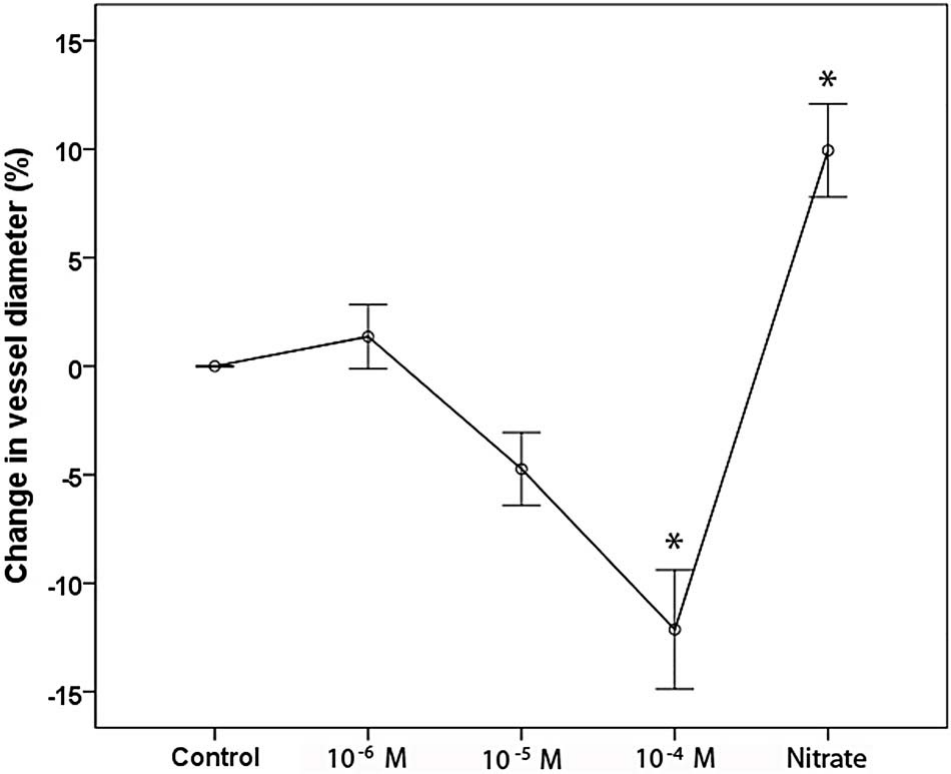
Mean vasomotor response to serial acetylcholine infusions and isosorbide dinitrate. There was significant vasoconstriction during the highest acetylcholine concentration whereas administration of isosorbide dinitrate caused vasodilation. n=31; *p<0.001 vs control.

The mean vessel response for each ACh infusion and isosorbide dinitrate injection are shown in figure 1. The mean response to incremental concentrations of ACh was progressive vasoconstriction, which was significant at the highest concentration (1.36±1.48%, −4.74±1.68% and −12.27±2.74% for 10^−6^, 10^−5^ and 10^−4^ M ACh, respectively, p<0.001 for 10^−4^ M ACh vs control). The mean response to isosorbide dinitrate was 9.94±2.15%, p<0.001 versus control. There was good agreement between the two segments analysed for each patient. The mean difference in endothelial function between the two segments analysed in each patient was 0.52 (SD 14.74) percentage points. Seven of 31 patients displayed vasoconstriction in one segment and vasodilation in the other segment. In such cases, the mean response was calculated. There was a biphasic response to ACh in 5 of 31 patients, with a vasodilatory response at 10^−6^ M of ACh (≥5% increase in vessel diameter) and vasoconstriction at 10^−4^ M ACh (≥5% decrease in vessel diameter).

### Adverse events

**Figure 2:**
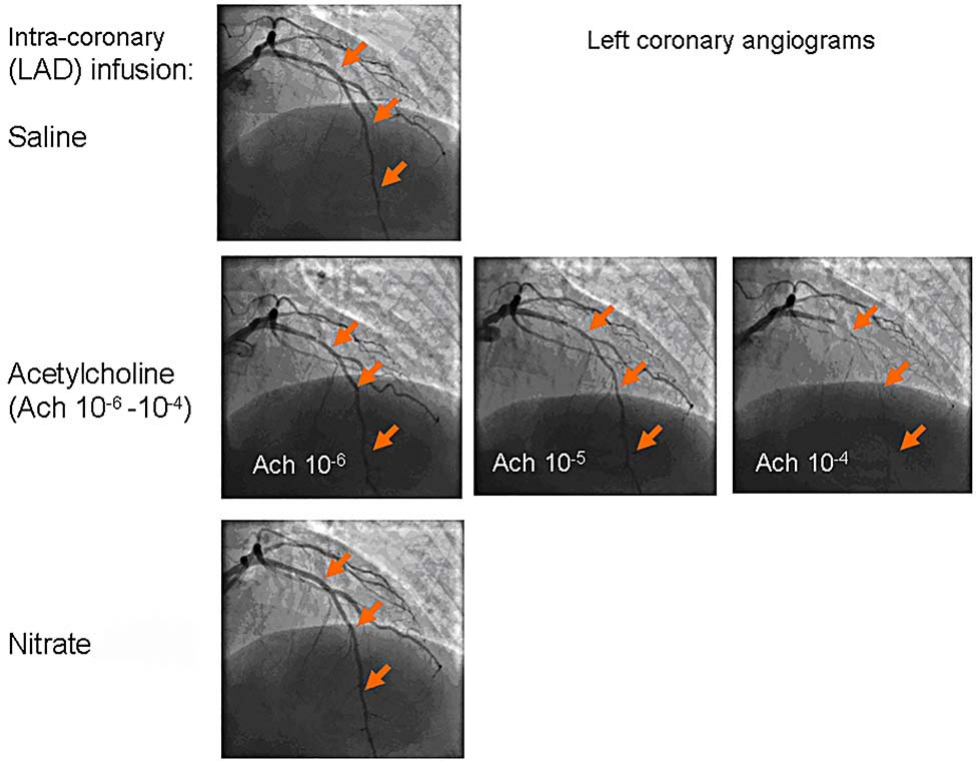
Angiographic example of severe coronary endothelial dysfunction. Progressive vasoconstriction of the left anterior descending artery after serial acetylcholine infusions. Isosorbide dinitrate injection caused complete reversal of vasoconstriction. LAD, left anterior descending.

Transient atrioventricular block was relatively common during ACh infusion (occurring in 5 patients), especially during the highest concentration, but this was always short-lived (less than 10 s) after the infusion was stopped. One patient developed transient atrial fibrillation. Clinical evidence of ischaemia was rare, occurring in only two patients. Figure 2 shows an example of severe vasoconstriction in the left anterior descending coronary artery in one patient who developed marked ischaemia during high-dose ACh infusion; this was quickly reversed with intracoronary nitrate injection. No serious or lasting complications were encountered.

### Correlations

**Figure 3:**
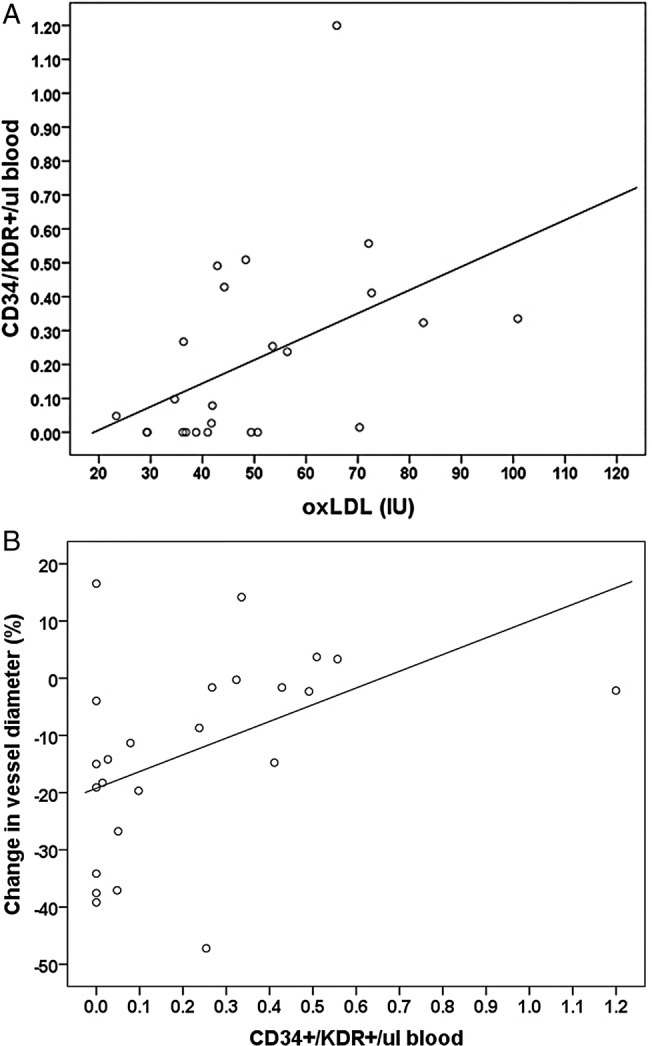
(A) Scatter plot of plasma oxLDL concentration and the number of circulating EPCs (CD34+/KDR+ cells). OxLDL was correlated with EPC numbers (n=24; r=0.46; p<0.05). (B) Scatter plot of the number of circulating EPCs (CD34+/KDR+ cells) and coronary endothelial function. Endothelial function is represented by % change in vessel diameter during acetylcholine, compared to control. EPC numbers were correlated with endothelial function (n=24; r=0.42; p<0.05). EPCs, endothelial progenitor cells; oxLDL, oxidised low-density lipoprotein.

The mean plasma oxLDL concentration was 47.2 IU and the mean number of circulating EPCs in venous blood was 0.21±0.06 per µL. There was a significant positive correlation between oxLDL and the number of circulating EPCs (r=0.46, p=0.02). The individual data for these results are displayed in figure 3A. Adding hypertensive status, hypercholesterolaemic status or gender individually to the model did not affect the relationship between oxLDL and EPCs (data not shown). Diabetes mellitus and smoking were present in no greater than three patients, so these variables were not included in the analysis due to insufficient power to detect an effect. One patient had a much greater value for EPCs and to ensure the regression was not unduly influenced by this value, a sensitivity analysis was conducted by was removing this data point from the model. Following this, the correlation between oxLDL and EPCs remained significant (p=0.02). There was also a significant positive correlation between the number of circulating EPCs and coronary endothelial function (r=0.42, p=0.04). This data are shown in figure 3B. Thus, patients with fewer circulating EPCs displayed more severe coronary endothelial dysfunction. Age inversely correlated with circulating EPC levels (r=−0.46, p=0.02). Given the potentially confounding effect of statins with regard to EPCs and endothelial function, an exploratory analysis was performed excluding three patients not prescribed a statin. After excluding these patients, the positive correlation between oxLDL and the number of circulating EPCs (r=0.43, p=0.04) and EPCs and endothelial function (r=0.53, p=0.01) remained significant. There was no significant correlation between oxLDL and coronary endothelial function (r=0.31, p=0.11). Endothelium-independent vasodilation induced by isosorbide dinitrate was not correlated with any parameter.

## Discussion

This study tested coronary endothelial function in patients with stable CHD and assessed its relationship with circulating levels of EPCs and oxLDL. The major novel findings of this study were as follows: (1) There was a high prevalence of coronary endothelial dysfunction in this patient population; (2) oxLDL correlated positively with EPCs; (3) EPCs correlated positively with coronary endothelial function.

### OxLDL and EPCs

The mean EPC count in the study population is broadly in agreement with previous studies.^17^ A novel finding was that oxLDL measured in the peripheral blood of patients with CHD was positively correlated with circulating EPC numbers. This result was contrary to much of the literature reporting in vitro toxicity of oxLDL toward EPCs. Besides the diverse methods to identify EPCs in the literature, our finding may be explained by a number of issues. In previous studies, EPC dysfunction occurred at 1–10 μg/mL oxLDL^11^ ^12^ and EPC apoptosis (5–10%) was induced only by 25 μg/mL oxLDL or higher.^9^ ^10^ ^18^ Wang *et al*^19^ reported a reduction in cultured EPCs (∼50%) at a concentration of 100 μg/mL oxLDL. However, in contrast, low concentrations (5 µg/mL) of oxLDL were found to have a positive effect on EPC tube formation through activation of endothelial nitric oxide synthase (eNOS).^20^ In our study, the mean plasma oxLDL concentration of 47.2 IU was equivalent to 14.1 μg/mL (personal communication from manufacturer). Furthermore, studies that investigated the protective effect of statins found that oxLDL-induced EPC dysfunction and senescence were profoundly inhibited by 1 μM atorvastatin, which may occur via activation of Akt.^11^ ^12^ Indeed, statin ‘reloading’ can be used to raise the numbers of circulating EPCs in patients undergoing PCI.^21^ Thus, the relatively low concentration of oxLDL in the blood and liberal use of statins in our contemporary CHD population may have protected circulating EPCs against oxLDL-induced damage. That circulating EPCs were actually increased in those with higher oxLDL concentration is a novel finding that merits further consideration and confirmatory study. We postulate that this positive correlation may be due to a host-repair response, induced by the damaging effects of increased circulating oxLDL on the vasculature; or that plasma oxLDL is a trigger (direct or indirect) for the release and mobilisation of EPCs from the bone marrow. OxLDL stimulates the release of several proinflammatory chemokines in patients with CHD^22^ and one or more of these factors may have been responsible for increased numbers of EPCs in the circulation. For instance, oxLDL markedly stimulates the release of interleukin (IL)-8 and growth regulated oncogene alpha (GRO-α) from peripheral blood mononuclear cells and platelets in patients with CHD.^22^ These family of IL-8/GRO-α chemokines have since been shown to promote the homing of EPCs to areas of ischaemic myocardium.^23^ The number of circulating EPCs may also be increased in acute coronary syndromes^4^ ^24^ and severe forms of CHD,^5^ which are both associated with elevated oxLDL in the blood.^25^ A recent study has found rapid intracoronary recruitment of EPCs in patients with ST elevation myocardial infarction, likely to represent a reparative response.^4^

### EPCs and endothelial function

We have shown that the number of circulating EPCs in the peripheral blood of patients with stable CHD was correlated with coronary endothelial function. Hill *et al*^26^ previously showed in 45 individuals without cardiovascular disease that peripheral vascular function (assessed by flow-mediated brachial artery reactivity) was correlated with the number of EC colony forming units in culture, thought at the time to represent EPCs derived from peripheral blood. It has since been established that this widely cited study assessed an aspect of endothelial biology that did not reflect the number of actual EPCs present in the circulation.^27^ EPCs are widely believed to originate from haematopoietic stem cells, which are positive for CD34 (or the more immature marker protein CD133) and the EC antigen, KDR. These putative EPCs appear to make a valuable contribution to vessel formation. Hence, the measurement of CD34+/KDR+ cells is thought to be the most appropriate way to define circulating EPCs,^27^ consistent with our study. Werner *et al*^28^ reported a positive correlation between EPCs and coronary endothelial function and their finding has not, to the best of our knowledge, been confirmed since by others. This study in 90 patients with stable CHD measured the number of circulating EPCs in peripheral blood using flow cytometry to quantify CD34+/KDR+ and CD133+ cells. The ability of EPCs to produce endothelial colony forming units was also measured. It was found using univariate analysis that patients with low EPC number had the most severely impaired coronary endothelial function and CD34+/KDR+ cells were more strongly correlated with endothelial function than CD133+ cells. The failure of endothelial colony forming units to independently predict endothelial function probably relates to their dissociation with the number of circulating CD34+/KDR+ cells.^27^ By improving the capacity of the endothelium to be repaired after vessel injury, an obvious link between circulating EPC numbers and coronary endothelial function exists. The confirmation herein and by Werner *et al*^28^ that lower numbers of EPCs are associated with impaired coronary endothelial function provides a pathological basis for the ability of EPCs to predict cardiovascular outcome.^29^

### Limitations

The method to identify EPCs in this study using specific haematopoietic and endothelial markers, CD34 and KDR, is consistent with contemporary guidance,^27^ but the most accurate way to quantify EPCs remains speculative. It may have been useful to assess EPC function, as this may have revealed a negative association with plasma oxLDL levels and provided insights into endothelial dysfunction present in patients with elevated oxidative stress. We did not measure oxLDL antibodies which may have provided an assessment of oxLDL formation over a longer time period, however, the correlation between these variables is not linear.^30^ Although, vasodilator drugs were withheld for 24 h before assessment of endothelial function, studies have shown that calcium channel blockers^31^ and ACE inhibitors^32^ can increase circulating EPCs. Thus, we cannot rule out the possibility that variable uptake of non-statin cardioprotective medications contributed to the correlations we found in our relatively small study.

## Conclusions

This observational study has confirmed a high prevalence of coronary endothelial dysfunction in patients with stable CHD. Plasma oxLDL correlated with the number of circulating EPCs in the blood, possibly due to the presence of a host-repair response and the protective effects of prescribed statins. Coronary endothelial function in this population was correlated with the number of circulating EPCs, which has been confirmed by one other group.^28^ Future studies should aim to define the reasons for reduced numbers of EPCs in cases of severe endothelial dysfunction and identify strategies to prevent this decline.
